# Deuteron Quadrupole Coupling Constants and Reorientation Correlation Times of Cations in Amino Acid Ionic Liquids

**DOI:** 10.1002/cphc.202500490

**Published:** 2025-10-26

**Authors:** David Kotwica, Dirk Michalik, Ralf Ludwig

**Affiliations:** ^1^ Institut für Chemie Abteilung für Physikalische Chemie Universität Rostock Albert‐Einstein‐Str. 27 18059 Rostock Germany; ^2^ Leibniz‐Institut für Katalyse e.V. Universität Rostock Albert‐Einstein‐Str. 29a 18059 Rostock Germany; ^3^ Department LL&M University of Rostock Albert‐Einstein‐Str. 25 18059 Rostock Germany

**Keywords:** amino acid ionic liquids, density functional theory calculations, dynamics, hydrogen bonding, NMR relaxation

## Abstract

The deuteron quadrupole coupling constants and reorientation correlation times of imidazolium cations in amino acid ionic liquids (AAILs) from NMR spin‐lattice relaxation times, *T*_1_, are determined. A proper determination of the reorientation correlation times *τ*_c_ of the acidic C(2)─H groups on the imidazolium rings requires knowledge of reliable NMR deuteron quadrupole coupling constants *χ*_D_. For that purpose, a relation between the deuteron quadrupole coupling constants *χ*_D_ and the proton chemical shifts *δ* ^1^H from density functional theory (DFT) calculated properties of differently sized clusters of the AAILs is first derived. Using this relation, the simple measurement of proton chemical shifts *δ* ^1^H provides an accurate estimate for *χ*_D_, which can then be used for determining the reorientation correlation times *τ*_c_. This method is applied to AAILs including the same imidazolium cation but differently functionalized anions. The obtained *χ*_D_ values range between 180 and 210 kHz, depending on the differently strong cation–anion interaction further enhanced by hydrogen bonding. The resulting reorientation correlation times *τ*_c_ indicate that the extreme narrowing condition is fulfilled for this type of IL. Using the Stokes–Einstein–Debye relation, the correlation times *τ*_c_ and the additionally measured viscosities *η* provide an estimate for the volume/size of the clusters present in solution.

## Graphical abstract

Connecting structure and dynamics. The deuteron quadrupole coupling constants and reorientation correlation times of the acidic C—H bond of imidazolium cations in amino acid ionic liquids are provided by using NMR property relationships and relaxation time experiments.

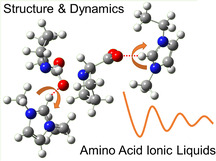

## 1 Introduction

In a pioneering work, Ohno et al. systematically synthesized amino acid ionic liquids (AAILs) using 1‐ethyl‐3‐methylimidazolium (EMIm^+^) as cation and 20 natural amino acids as anions.^[^ ^1^, ^2^ ^]^ Compared to other classes of ionic liquids (ILs), AAILs offer additional desirable properties of being biodegradable, nontoxic, and inexpensive.^[^ ^3^, ^4^ ^]^ Due to the controllable balance of Coulomb interaction, hydrogen bonding, and dispersion forces, the physicochemical properties of ILs can be tailored for a wide range of potential applications.^[^ ^5^, ^6^, ^7^ ^]^ This is particularly true for AAILs simultaneously containing proton donor and acceptor functions on the anion such as carboxy and amine groups allowing for the formation of H─bond networks among the molecular ions.^[^ ^8^, ^9^, ^10^, ^11^, ^12^, ^13^ ^]^

**Scheme 1:**
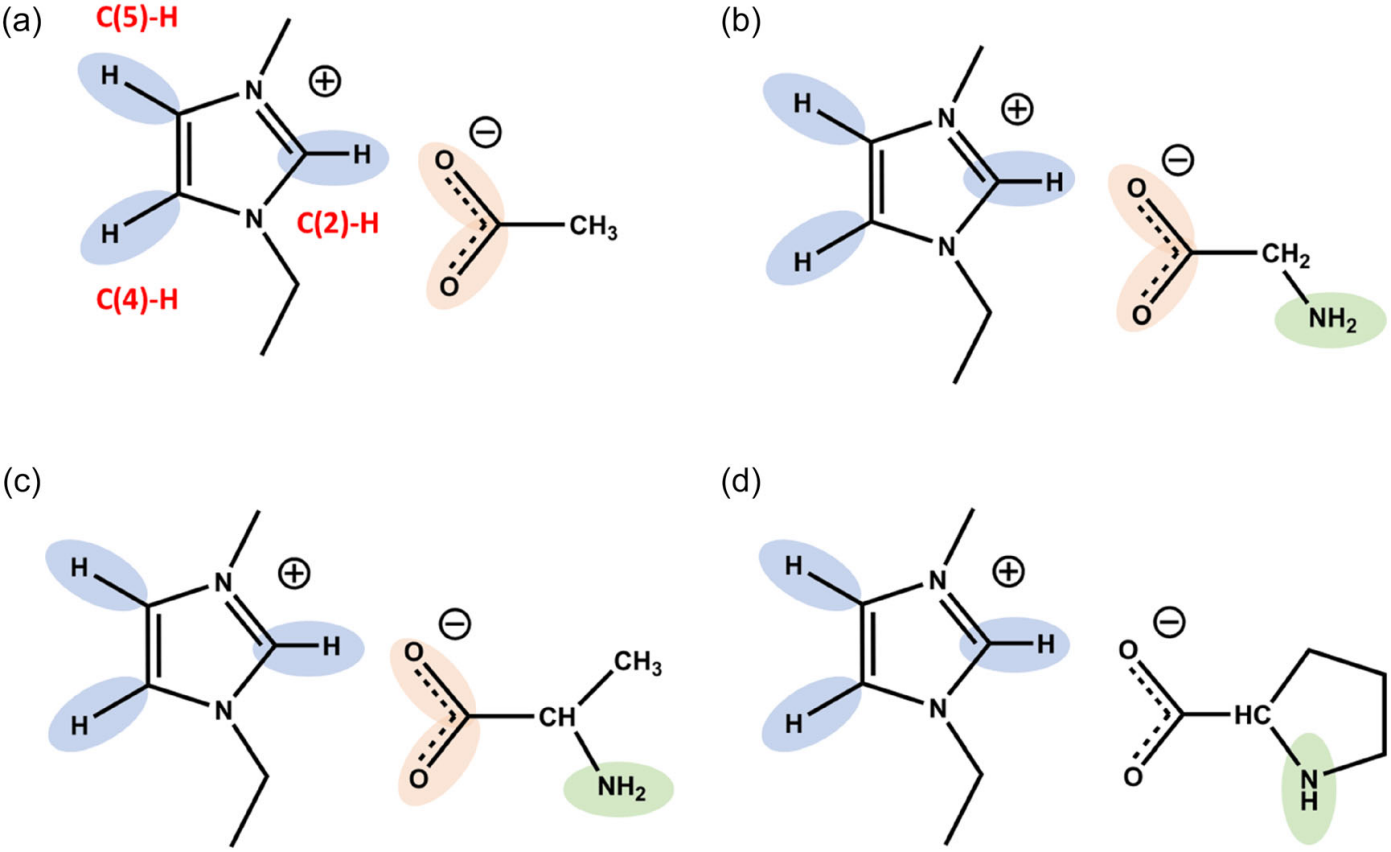
We investigated the IL a) 1‐ethyl‐3‐methylimidazolium acetate [EMIm][OAc] and the AAILs b) 1‐ethyl‐3‐methylimidazolium glycinate [EMIm][Gly], c) 1‐ethyl‐3‐methylimidazolium alaninate [EMIm][Ala], and d) 1‐ethyl‐3‐methylimidazolium prolinate [EMIm][Pro]. All ILs provide the proton donor abilities at the imidazolium cation (blue) and acceptor abilities at the carboxylate group of the anions (red). The AAILs [EMIm][Gly] and [EMIm][Ala] have an additional amine group and the IL [EMIm][Pro] an additional amide group as proton donors (all green). For the 1‐ethyl‐3‐methylimidazolium cation, the C(2)─H, C(4)─H and C(5)─H bonds at the ring are indicated (red).

Recently, we reported spectroscopic evidence for the existence of anionic dimers in imidazolium‐based AAILs.^[^ ^14^ ^]^ In the simplest AAIL [EMIm][Gly], two glycinate anions form two hydrogen bonds between either the carboxylate and amino groups allowing for attractive anion–anion interaction despite the Coulomb repulsion between the ions of like charge. Far infrared spectra showed spectral features of H─bonds between cations and anions (c‐a) in the IL [EMIm][OAc] and the AAIL [EMIm][Gly] but additional spectral signatures for the latter assigned to H─bonds between anion and anion (a‐a). Obviously, we find solely H─bonded ion pairs (c‐a) for [EMIm][OAc], whereas in the AAIL [EMIm][Gly], H─bond networks of kind (c‐a‐a‐c) are formed because the AAIL anions not only provide two proton acceptors at the carboxylate group but also two proton donors at the amine group. Such a network formation should have strong influence on the dynamics of the IL constituents. For that purpose, we studied the rotational dynamics of the imidazolium cations in the IL 1‐ethyl‐3‐methylimidazolium acetate [EMIm][OAc] and the AAILs 1‐ethyl‐3‐methylimidazolium aminoacetate (glycinate), [EMIm][Gly], 1‐ethyl‐3‐methylimidazolium (S)‐2‐aminopropionate (alaninate) [EMIm][Ala], and 1‐ethyl‐3‐methylimidazolium prolinate [EMIm]Pro], respectively (see **Scheme** **1**). We measured NMR deuteron spin‐lattice relaxation times *T*_1_ of the acetic C(2)—D bonds on the imidazolium rings of the cations. Reliable deuteron quadrupole coupling constants *χ*_D_ of the C—D group at the ring system of imidazolium cations are required for the evaluation of the correlation times, which unfortunately cannot be measured directly in the liquid state. For that reason, we determined the deuteron quadrupole coupling constants *χ*_D_ for AAILs, using a relation between the NMR chemical shifts *δ* ^1^H and quadrupole coupling constants *χ*_D_, which both are sensitive probes of hydrogen bonding as shown for molecular and ILs as well.^[^ ^15^, ^16^ ^]^ This has been in particular shown for water, the archetype of hydrogen bonded liquids. From the solid state to the gas phase of the unique liquid, *δ* ^1^H changes by one ppm and *χ*_D_ by almost 90 kHz ranging from 220 kHz in ice up to 308 kHz in the gas phase.^[^ ^17^, ^18^, ^19^, ^20^ ^]^ These NMR properties appear to depend strongly on temperature, pressure or polarity, even for ILs characterized by a mixture of Coulomb interaction, hydrogen bonding, and dispersion forces.^[^ ^9^, ^11^, ^12^, ^21^ ^]^ Usually, molecular C—H bonds weakly depend on hydrogen bonding and only negligible changes in the spectroscopic properties are reported in the literature.^[^ ^22^, ^23^ ^]^ That is different for the ring C—H bonds of imidazolium cations in the AAILs, which are extremely sensitive to the chemical environment resulting in ion pair formation, strong acidity and even decomposition. This applies in particular to the acidic C(2)─H group on the imidazolium ring, which is known to form weak to strong H─bonds depending on the strength of the attractive Coulomb interaction between cation and anion.^[^ ^24^, ^25^ ^]^

Herein, we show that NMR chemical shifts *δ* ^1^H, and quadrupole coupling constants *χ*_D_ for C—H bonds in imidazolium based AAILs appear to reflect a similar type of electronic disorder caused by hydrogen bonding. Relationships between proton chemical shifts and deuteron quadrupole coupling constants were computed for clusters of the IL 1‐ethyl‐3‐methylimidazolium acetate [EMIm][OAc] and the AAILs 1‐ethyl‐3‐methylimidazolium aminoacetate (glycinate), [EMIm][Gly], 1‐ethyl‐3‐methylimidazolium (S)‐2‐aminopropionate (alaninate) [EMIm][Ala], and 1‐ethyl‐3‐methylimidazolium prolinate [EMIm]Pro] including *n *= 1‐6 ion pairs. These relationships can be used for deriving structural and spectroscopic properties which are not available for the liquid phase. With these relationships, we are able to reliably evaluate and interpret measured NMR deuteron relaxation times (1/*T*_1_)_D_. Using the Stokes–Einstein–Debye (SED) relationship, the correlation times $\tau_{c}$ and the additionally measured viscosities *η*, the volume/size of the clusters in solution can be estimated and discussed with regard to the structures present in the ILs.

## 2 Experimental Section

### 2.1 Materials and Sample Preparation

The IL [EMIm][OAc] and the AAILs [EMIm][Gly], [EMIm][Ala], and [EMIm][Pro] have been purchased from IoLiTec (Ionic Liquids Technologies GmbH, Heilbronn) with a purity of about 96%. We characterized the ILs by standard liquid NMR and IR spectroscopy. The AAILs have been further characterized by elemental analysis because a Karl‐Fischer titration was not applicable. For the IL [EMIm][OAc], we measured a water concentration of 150 ppm.^[^ ^14^ ^]^

Sample preparation for the NMR experiments was performed in the following manner. The ILs were loaded into a glass tube (5 mm outer diameter; 20 mm long) and connected to a high vacuum grade valve (HI‐VAC). All manipulations were performed in N_2_ atmosphere. The sample was then attached to a vacuum line, and the nitrogen was pumped off under vacuum to a final pressure above the sample of 2 × 10^−5^ mPa. To fully degas the material the sample was connected to vacuum line while being heated. After degassing, the neck of the tube was sealed off. The sealed sample was then transferred into an outer NMR tube with deuterated DMSO in the external tube NMR probe for analysis with ^2^H NMR spectroscopy.

### 2.2 NMR Chemical Shift and Relaxation Time Measurements

The ^1^H NMR spectra of the neat AAILs were recorded on a Bruker 250 MHz spectrometer using a 4 mm probe within a 5 mm outer sample tube. Temperature calibrations were carried out using the ethylene glycol NMR thermometer.^[^ ^26^ ^]^ The *δ* ^1^H spectra of [EMIm][OAc], [EMIm][Gly], [EMIm][Ala], and [EMIm][Pro] are analyzed for the chemical shift range between 7 ppm and 11 ppm, wherein we observe the NMR signals of the imidazolium ring protons.^[^ ^21^ ^]^ For all ILs, the C(2)─H protons are about 2 ppm stronger downfield shifted than the C(4)─H and C(5)─H protons due to the strong acidity of this bond, also reflected in stronger C—H read shifts in the IR spectra (for definition of the C—H bonds see Scheme 1).^[^ ^22^ ^]^ It is also notable that the *δ* ^1^H chemical shifts of the IL [EMIm][OAc] are the stronger downfield shifted than those of the AAILs which themselves only differ slightly. The (c‐a) H─bond in [EMIm][OAc] appears to be stronger, as the significant charge transfer from the OAc^‐^ anion to the C—H antibond orbitals of the EMIm^+^ cation cannot be compensated by charge transfer to the N—H antibond orbitals of the anion itself.^[^ ^27^, ^28^, ^29^, ^30^ ^]^

The ILs were then deuterated by ^1^H/^2^H exchange in ^2^H_2_O and properly dried. ^1^H NMR spectra confirmed that exchange took only place mainly at the C(2)─H position. Longitudinal ^2^ H relaxation times *T*_1_ were measured using a BRUKER Avance 250 spectrometer at a resonance frequency of *ν* = *ω*/2*π* = 38.35 MHz, using the inversion recovery (180°–*τ*–90°) pulse sequence. The *T*_1_ relaxation times are estimated to be accurate to within ±2%.

### 2.3 DFT Calculations of IL and AAIL Clusters

**Figure 1:**
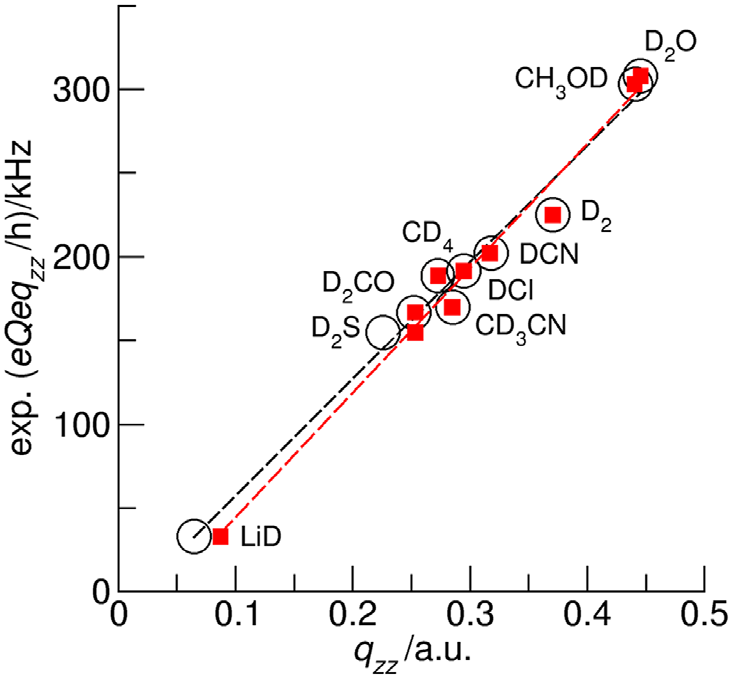
Experimental quadrupole coupling constants plotted versus B3LYP/6‐31+G* (open circles) and B3LYP‐D3/6‐31+G* (filled squares) calculated electric field gradients for deuteron nuclei in small molecules such as LiD, CD_4_, DCN, and D_2_O following the approach by Huber.^[^ ^35^ ^]^ The slopes provide reasonable nuclear quadrupole moments for the applied levels of theory and can be used for calculating deuteron quadrupole coupling constants.

We optimized varies sized clusters of the AAILs 1‐ethyl‐3‐methylimidazolium acetate [EMIm][OAc], 1‐ethyl‐3‐methylimidazolium aminoacetate (glycinate) [EMIm][Gly], 1‐ethyl‐3‐methylimidazolium (S)‐2‐aminopropionate (alaninate) [EMIm][Ala], and 1‐ethyl‐3‐methylimidazolium prolinate [EMIm][Pro] by means of density functional theory (DFT) calculations at the B3LYP/6‐31+G* and B3LYP‐D3/6‐31+G* theory levels using the Gaussian 09 program.^[^ ^31^, ^32^, ^33^, ^34^ ^]^ The proton chemical shifts *δ* ^1^H and the deuteron quadrupole coupling constants *χ*_D_ were then calculated using the optimized geometries obtained with the same DFT methods and basis sets. The NMR proton chemical shifts *δ* ^1^H were referenced against the standard of TMS (chemical shielding 32.08 ppm). The deuteron quadrupole coupling constants *χ*_D_ = (*eQeq*_zz_/*h*) were obtained by multiplying the calculated main components of the electric field gradients *eq*_zz_ with a calibrated nuclear quadrupole moment *eQ*, which results from plotting the gas phase quadrupole coupling constants from microwave spectroscopy versus the calculated main components of the electric field gradients for small molecules such as LiD, CD_4_, DCN, and D_2_O following the approach by Huber et al.^[^ ^35^, ^36^ ^]^ We obtained a linear relation *χ*_D_ = −12.659 + 696.7*eq_zz_, which can be used for calculating deuteron quadrupole coupling constants *χ*_D_ at the B3LYP/6‐31+G* level of theory, no matter whether we study gas phase molecules, molecular associates, or IL clusters. However, it is well known in the literature that DFT methods do not properly take dispersion interaction into account.^[^ ^32^, ^33^, ^34^ ^]^ For that reason, we used Grimme's D3 approach also for deriving a new calibrated nuclear quadrupole moment. Including dispersion interaction changes, in particular, the geometries of the molecules LiH and H_2_S resulting in higher calculated electric field gradients, *eq*_zz_, and a slightly different fit *χ*_D _= −30.637 + 745.0*eq_zz_, which should be then used for calculating *χ*_D_ values at the B3LYP‐D3/6‐31+G* level. The two plots are shown in **Figure** **1** for comparison.

### 2.4 Viscosity Measurements

The viscosities were measured of the IL [EMIm][OAc] and the AAILs [EMIm][Gly], [EMIm][Ala], and [EMIm][Pro] in the temperature range between 288 and 343 K using a rolling‐ball viscometer Lovis 2000 M/ME from Anton Paar with accuracy of *δη*/*η* = ±0.5% and *δT* = ±0.02 K (according to Anton Paar Technical Manual).^[^ ^37^ ^]^

#### 2.4.1 Results and Discussion

**Figure 2:**
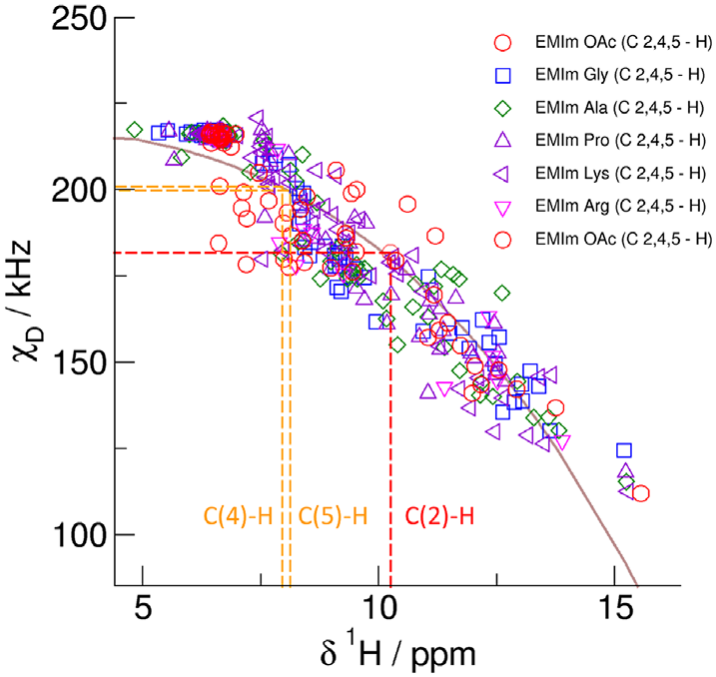
Deuteron quadrupole coupling constants *χ*_D_ of the C(2)‐D, C(4)‐D, and C(5)‐D deuterons plotted versus chemical shifts *δ* ^1^H of the C(2)─H, C(4)─H, and C(5)─H protons of the 1‐ethyl‐3‐methylimidazolium cations as present in the IL [EMIm][OAc] and the AAILs [EMIm][Gly], [EMIm][Ala], and [EMIm][Pro]. Both NMR properties were calculated for clusters *n* = 1‐6 at the B3LYP‐D3/6‐31+G* level of theory. The relation between *χ*_D_ and *δ* ^1^H allows to predict reliable deuteron quadrupole coupling constants if proton chemical shifts are available from experiment. This is demonstrated for the IL [EMIm][OAc] (see dashed red and orange lines).

The proton chemical shifts *δ* ^1^H chemical shifts and the deuteron quadrupole coupling constant *χ*_D_ were calculated for each C—H/D bond on the imidazolium cations at the minimum geometry for all IL complexes. Both NMR spectroscopic properties reflect the chemical environment experienced by the various C—H bonds. If C—H bonds are not interacting with the counterions, typical gas‐phase values are observed. In contrast, strong interaction between the C—H bonds and the anion results in downfield chemical shifts *δ* ^1^H and smaller quadrupole coupling constants *χ*_D_. Cooperative effects in larger complexes may even lead to spectroscopic properties as known for the liquid state. The calculated spectroscopic properties are all shown in **Figure** **2**. The deuteron quadrupole coupling constants *χ*_D_ are plotted versus *δ* ^1^H chemical shifts which themselves are referenced to TMS. A cubic relationship is obtained between both spectroscopic properties.

(1) $$\chi_{D}=A\text{-}B{\left(\delta^{1}H\right)}^{3}\;\mathrm{with}\;A=217.8\;\mathrm{kHz}\;\mathrm{and}\;B=0.03366\;\mathrm{kHz}/{\mathrm{ppm}}^{3}$$

**Figure 3:**
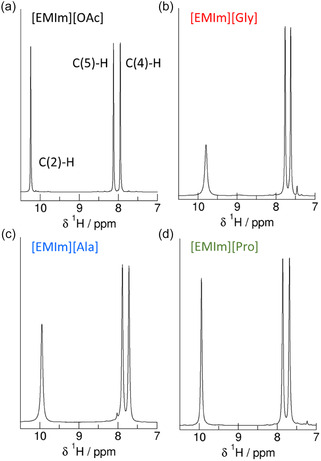
Measured proton chemical shifts *δ* ^1^H of the C(2)─H, C(4)─H, and C(5)─H protons of the 1‐ethyl‐3‐methylimidazolium cations as present in a) [EMIm][OAc], b) [EMIm][Gly], c) [EMIm][Ala], and d) [EMIm][Pro]. The *δ* ^1^H values of the IL [EMIm][OAc] are shown in Figure 2 for demonstrating how to predict reliable deuteron quadrupole coupling constants.

**Figure 4:**
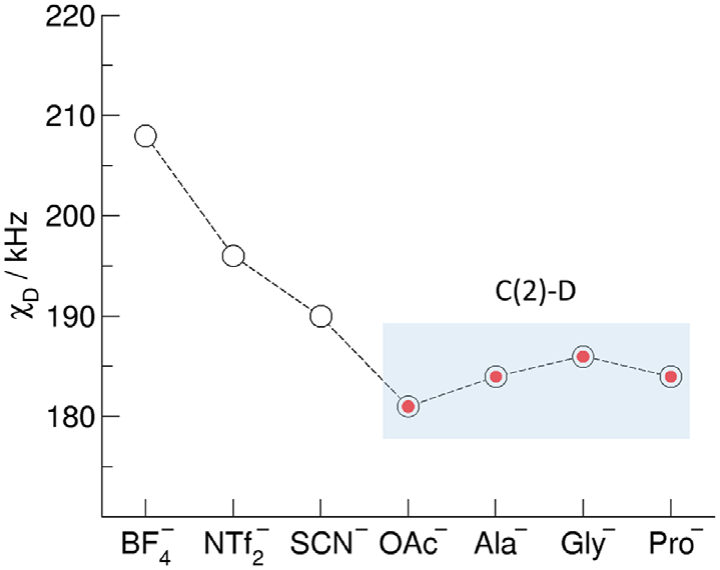
Deuteron quadrupole coupling constants *χ*_D_ of the C(2)‐D deuterons at the imidazolium cations as obtained from measured proton chemical shifts *δ* ^1^H and the calculated relation between both properties as shown in Figure 2. For comparison, we show the corresponding *χ*_D_ values for other ILs determined using the same approach.^[^ ^53^ ^]^

**Figure 5:**
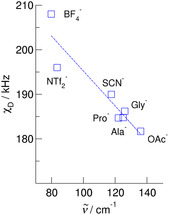
Deuteron quadrupole coupling constants *χ*_D_ of the C(2)‐D deuterons at the imidazolium cations plotted versus far infrared frequencies *ν*_σ_, describing the interaction strength between cation and anion along the C(2)─H bond.^[^ ^55^, ^56^ ^]^

Consequently, the chemical shifts *δ* ^1^H may be used as an accurate indirect measure of the deuteron quadrupole coupling constant *χ*_D_. The *δ* ^1^H spectra of all ILs considered in this study are shown in **Figure** **3**. They include the NMR signals of the imidazolium ring protons ranging between 7 and 11 ppm. In Figure 2, we show how we derived the *χ*_D_ values for the C(2)—D, C(4)—D, and C(5)—D deuterons on the imidazolium ring in [EMIm][OAc] from the measured chemical shifts *δ* ^1^H by using the relationship between the two NMR properties given in Equation (1). Proton chemical shifts of 10.24 ppm for C(2)─H, 7.95 ppm for C(4)─H, and 8.12 ppm for C(5)─H deliver *χ*_D_ values of about 181.7, 200.9, and 199.8 kHz in this sequence. The stronger H─bond ^+^C(2)─H^**…**^OOC^−^ lead to 20 kHz smaller *χ*_D_ values compared to the weakly bound (C4,5)─H bonds. It is also shown that the chemical shifts are more sensitive to H─bonding than the deuteron quadrupole coupling constants. In **Figure** **4**, we show the derived *χ*_D_ values for the C(2)—D groups as obtained for [EMIm][OAc] and the three AAILs. For comparison, we also show the *χ*_D_ values for other imidazolium‐based ILs using the same method but different anions BF_4_^−^, NTf_2_^−^, and SCN^−^. The values of *χ*_D_ range from 208 kHz down to 181 kHz in the order of increasing interaction strength between cation and anion via the acidic C(2)─H bond. Although less sensitive to H─bonding than the proton chemical shifts *δ* ^1^H, the deuteron quadrupole coupling constants *χ*_D_ are a sensitive probe for describing the H─bond enhanced interaction between cation and anion in ILs. This is demonstrated in **Figure** **5**, wherein we plotted the *χ*_D_ values versus far infrared frequencies *ν*_σ_, which are known to be a suitable measure of the cation–anion interaction (C(2)─H^…^A).^[^ ^38^, ^39^ ^]^ We obtain a reasonable linear relation: Stronger interaction results in lower *χ*_D_ values and higher frequencies *ν*_σ_. The larger deviation from the obtained fit in the frequency range between 80 cm^−1^ and 100 cm^−1^ results from the fact that the *ν*_σ_ values not only depend on the force constants but as well as reduced masses following the equation for the harmonic oscillator $\tilde{\nu }=1/2\pi c{\left(k/\mu \right)}^{1/2}$, where *c* is the speed of light, *k* the force constant and *μ* the reduced mass. The two values refer to the ILs including BF_4_^−^ and NTf_2_^−^ anions which differ significantly in mass.

Using the derived deuteron quadrupole coupling constant *χ*_D_, we can now determine the C(2)─H rotational correlation times *τ*_*c*_ from ^2^H longitudinal relaxation times, *T*_1_. The ^2^H nuclear magnetic relaxation results from the interaction of the nuclear quadrupole moment with the electric field gradient at the ^2^H‐nucleus, which is caused by the electron distribution surrounding the nucleus along the C(2)—D bond. The deuteron relaxation rate (1/*T*_1_)_*D*_ is then given by^[^ ^40^, ^41^ ^]^

(2) $${\left(\frac{1}{T_{1}}\right)}_{D}=\frac{3}{10}\pi^{2}\left(1+\frac{\eta_{D}^{2}}{3}\right)\chi_{D}^{2}\;\left\{\frac{\tau_{c}}{1+\omega_{0}^{2}\tau_{c}}+\frac{4\tau_{c}}{1+4\omega_{0}^{2}\tau_{2}^{c}}\right\}$$

where *χ*_D_ is the ^2^H nuclear quadrupole coupling constant, *η*_D_ is the corresponding asymmetry parameter, and *τ*_c_ is the rotational correlation time.

In molecular liquids, providing low viscosities, the extreme‐narrowing condition *ω*_0_τ_c_ << 1 is usually fulfilled. However, this is not necessarily the case for ILs, as there is evidence of extremely stretched orientation dynamics, which can extend the spectral densities in Equation (2) up to the Larmor frequency.^[^ ^42^, ^43^ ^]^

Consequently, we have carefully checked that the relaxation time experiments for all AAILs were performed in the extreme narrowing limit. We will see later that the largest correlation times *τ*_*c*_ at 303 K never succeed five hundred picoseconds. Calculating the product *ω*_0_ *τ*_c_ (36.35·10^6^ Hz × 500·10^−12^ s ≈ 0.018) is smaller than 1 and supports the extreme narrowing condition. In such a case, Equation (2) reduces to

(3) $${\left(\frac{1}{T_{1}}\right)}_{D}=\frac{3}{10}\pi^{2}\left(1+\frac{\eta_{D}^{2}}{3}\right)\chi_{D}^{2}\;\tau_{c}$$

Equation (3) shows that we have access to the correlation times *τ*_c_, if the deuteron quadrupole coupling constant *χ*_D_ and the asymmetry parameter *η* are known. The asymmetry parameters *η* are usually small and can be neglected here.^[^ ^16^ ^]^ Thus, we have direct access to the correlation times *τ*_*c*_.

**Figure 6:**
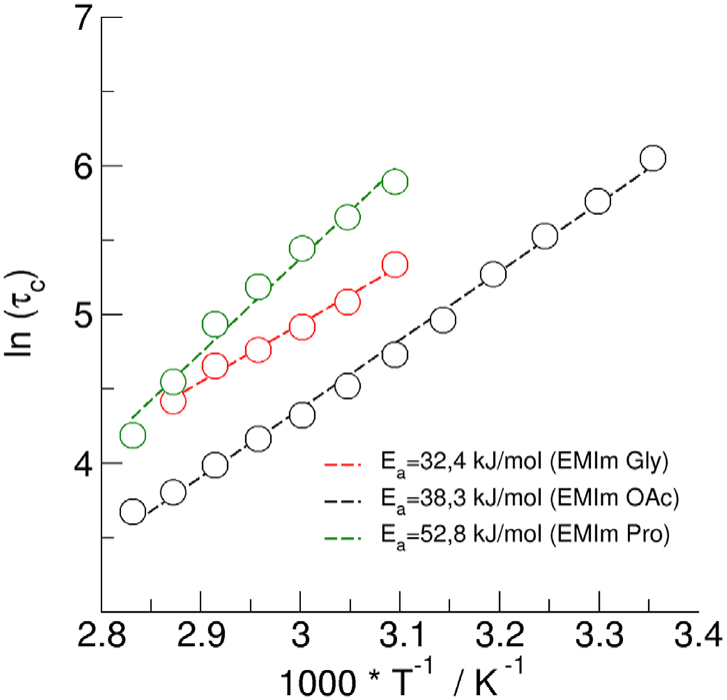
Rotational correlations times *τ*_c_ of the C(2)—D bonds in ps of the EMIm cations in the IL [EMIm][OAc] (circles) and the AAILs [EMIm][Gly] (squares) and [EMIm][Pro] (diamonds) in the temperature range between 293 and 353 K. The data for the AAIL [EMIm][Ala] could not be shown as described in the text.

As described above, we adapted an idea by Wendt and Farrar for deriving reliable *χ*_D_ values in the liquid phase.^[^ ^44^, ^45^, ^46^ ^]^ They could show for pure methanol, as well as for methanol in mixtures with tetrachloromethane, that the correlation time for the reorientation of the O—H bond can be derived from the spin‐lattice relaxation rate for the deuteron [Equation (3)]. Due to a strong coupling parameter, the deuteron quadrupole relaxation is entirely an intramolecular process. Other relaxation mechanisms or the separation of intra‐ and intermolecular relaxation contributions do not have to be considered. To derive coupling parameters for determining reliable rotational correlation times, the above given relationship of the NMR spectroscopic properties [Equation (3)] is very helpful. While the quadrupole coupling constants *χ*_D_ are generally not available for imidazolium‐based ILs, measuring the chemical shift of the C—H protons *δ* ^1^H of the ILs [EMIm][OAc], [EMIm][Gly], [EMIm][Ala], and [EMIm][Pro], is not a challenge. The C(2)─H proton chemical shifts range from 10.24 ppm for [EMIm][OAc] up to 9.94 ppm for [EMIm][Pro], suggesting the strongest H─bonding for the first IL. Using these chemical shifts *δ* ^1^H, we obtain corresponding quadrupole coupling constants *χ*_D_ from the given relationship between the NMR properties. The measured chemical shifts *δ* ^1^H and the derived quadrupole coupling constant *χ*_D_ are listed in **Table** **1**. We measured the NMR deuteron relaxation times for C(2)—D in the EMIm^+^ cations of [EMIm][OAc], [EMIm][Gly], [EMIm][Ala], and [EMIm][Pro], respectively. Using the relation between *δ* ^1^H und *χ*_D_, we finally determined the rotational correlation times *τ*_c_ as a function of temperature (see **Figure** **6**). Unfortunately, we could not derive *τ*_c_ for [EMIm][Ala] because of too fast H/D exchange between different sites on the NMR time scale. Thus, the average correlation times are not describing the motion of the C(2)─H molecular vector at the EMIm^+^ cation properly. The *τ*_c_ values increase in the order [EMIm][OAc], [EMIm][Gly], and [EMIm][Pro]. That [EMIm][OAc] shows the shortest correlation times might be a bit surprising because recently measured far IR spectra suggest that the cation–anion interaction is the strongest for the ILs considered here. The reason is the formation of quasi ion pairs between the acidic C(2)─H of the imidazolium cation and the carboxylate group of the counterion. In contrast, the AAILs include additional functional groups NH_2_ and NH allowing the formation of H─bond networks across all ions. Recently, we reported for [EMIm][Gly] the formation of anionic dimers with two strong H─bonds between the ions of like charge.^[^ ^14^ ^]^ Obviously extended H─bond networks result in longer correlation times. This behavior is also observed for the viscosities as shown later.

**Table 1:** Measured proton chemical shifts *δ* ^1^H of the C(2)─H, C(4)─H, and C(5)─H protons on the 1‐ethyl‐3‐methylimidazolium cations as present in [EMIm][OAc], [EMIm][Gly], [EMIm][Ala], and [EMIm][Pro] and the corresponding quadrupole coupling constants derived from the relation in Figure 2.

C(X)─H	EMIm‐ILs	*δ*1H [ppm]	*χ*_D_ [kHz]
X = 2	OAc	10.24	181.7
	Gly	9.79	186.3
	Pro	9.94	184.8
	Ala	9.94	184.8
X = 4	OAc	8.12	199.8
	Gly	7.77	202.1
	Pro	7.86	201.5
	Ala	7.89	201.4
X = 5	OAc	7.95	201.0
	Gly	7.62	203.0
	Pro	7.69	202.6
	Ala	7.72	202.4

The correlations times *τ*_c_ could be reasonably described by Arrhenius behavior. We obtained activation energies of about 38.3 kJ mol^−1^ for [EMIm][AOc], 32,4 kJ mol^−1^ for [EMIm][Gly], and 52,8 3 KJ mol^−1^ for [EMIm][Pro]. Considering additionally the activation energies for [EMIm][DCA], [EMIm][NTf_2_], and [EMIm][SCN] from the literature suggest that *E*_A_ constantly increases with the interaction strength of the anion and on top with additional functional groups on the anion. For completeness, we also applied the Vogel–Fulcher–Tammann (VFT) approach providing temperature dependent activation energies. As the VFT contains an additional parameter, the adjustment looks better, but only changes the result slightly.

**Figure 7:**
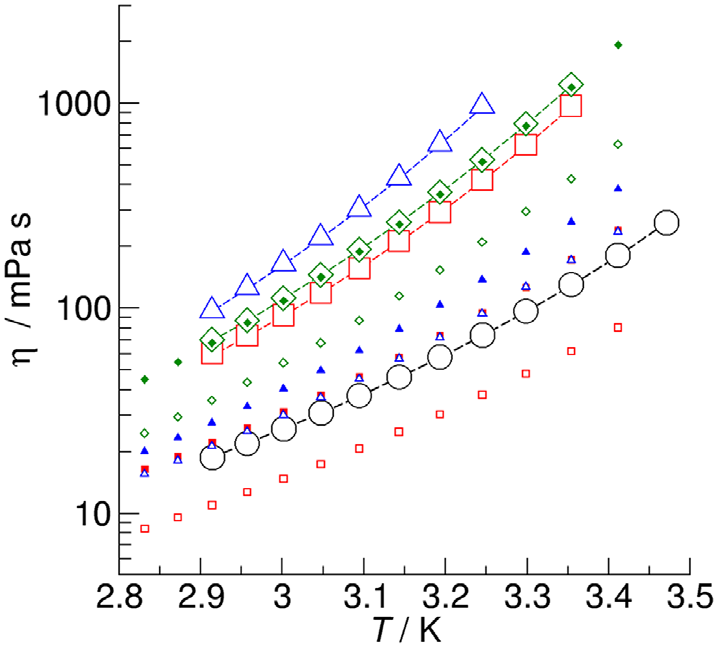
Viscosities *η* in mPa s of the IL [EMIm][OAc] (circles) and the AAILs [EMIm][Gly] (squares), [EMIm][Ala] (triangles), and [EMIm][Pro] (diamonds) in the temperature range between 288 and 343 K. We added AAIL viscosities measured by Gouveia et al.^[^ ^47^ ^]^ (small filled symbols) and Muhammad et al.^[^ ^48^ ^]^ (small open symbols).

The same applies to the viscosities that we measured for [EMIm][OAc], [EMIm][Gly], [EMIm][Ala], and [EMIm][Pro] respectively. As shown in **Figure** **7**, the viscosities exhibit the same order as the corresponding correlation times. For the AAIL [EMIm][Pro], the viscosities were in perfect agreement with those measured by Gouveia.^[^ ^47^ ^]^ In contrast, the viscosities of the other ILs were higher than those reported in the literature.^[^ ^48^, ^49^ ^]^ It is well known in the literature that even small impurities with water can significantly reduce the viscosities. We therefore assume that the highest measured values are the most reliable. It is notable that the activation energies for the correlation times and the viscosities of [EMIm][OAc] (38.3 versus 38.9 KJ mol^−1^) and [EMIm][Pro] (52.8 versus 53.9 KJ mol^−1^) are almost the same magnitude, indicating that the molecular rotational correlation times *τ*_c_ and the macroscopic viscosities *η* describe similar motion.

We know check the validity of the SED relation, which is derived from classical hydrodynamics and simple kinetic theory.^[^ ^50^, ^51^ ^]^ In the SED relation [Equation (4)]

(4) $$\tau_{c}=\frac{V_{\mathrm{eff}}}{k_{B}T}\eta$$

**Figure 8:**
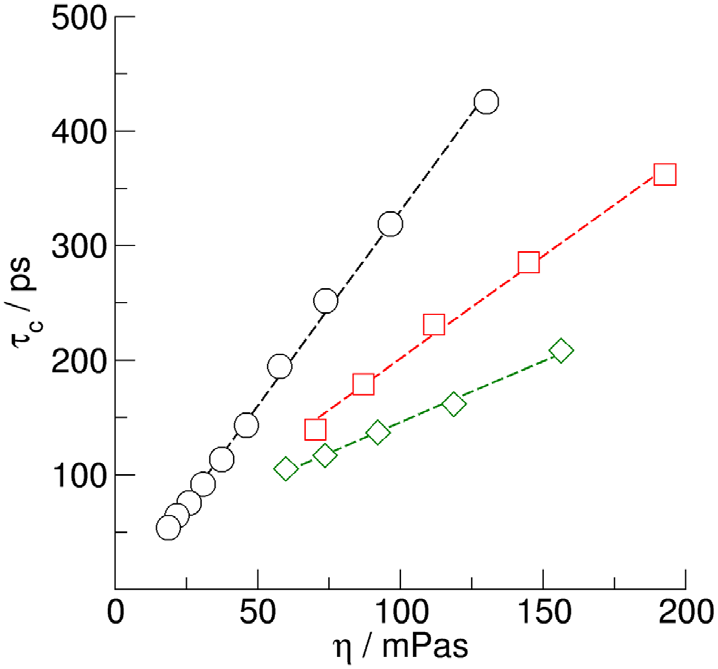
Correlation times *τ*_c_ plotted versus viscosities *η*. According to the SED relation, the linear behavior indicates the presence of an effective volume *V*_eff_ for the entire temperature range.

**Figure 9:**
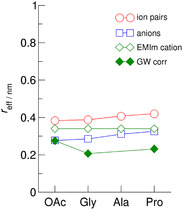
Effective radii *r*_eff_ of the ILs [EMIm][OAc], [EMIm][Gly], and [EMIm][Pro] determined from the rotational correlation times *τ*_c_ and viscosities *η* by using the SED relation. It is clearly seen that the resulting *r*_eff_ values are smaller than the calculated radii of ion pairs and even smaller than those of the cation EMIm^+^ and the anions OAc^−^, Gly^−^, and Pro^−^. The best agreement is found for the IL [EMIm][OAc], wherein “quasi” ion pairs are formed.

τ_c_ is the reorientation correlation time, *η* is the viscosity, *k*_*B*_ is the Boltzmann constant, *T* is the temperature, and *V*_eff_ is the effective volume. If we plot τ_c_ versus *η* for [EMIm][OAc], [EMIm][Gly], and [EMIm][Pro], we observe linear behavior (see **Figure** **8**). *V*_eff_ is then obtained by multiplying the volume *V* with the so‐called Gierer–Wirtz factor *f*.^[^ ^52^, ^53^, ^54^ ^]^ For neat liquids, *f* has a value of 1/6 ≈ 0.16. The friction factor is only 16% of the value under so‐called stick‐conditions, and we reach the slip limit. From the slope of the plot in Figure 8, we can estimate the effective volumes *V*_eff_ to be about 0.0875 nm^3^ for [EMIm][OAc], 0.0371 nm^3^ for [EMIm][Gly], and 0.0523 nm^3^ for [EMIm][Pro]. If we assume a spherical shape of the solute particles (the cations or ion pairs), we obtain an effective radii *r*_eff_ of about 0.275, 0.207, and 0.232 nm. Despite correcting the friction factor, the obtained radii from the SED relation are still below the calculated values for the cations, anions and ion pairs present in the ILs.^[^ ^31^ ^]^ As shown in **Figure** **9**, the best agreement is achieved for [EMIm][OAc], for which we observed ‘quasi’ ion pairs with specific H─bonds only between cation and anion. For the AAILs characterized by H─bond networks across the ions, the SED does not describe the situation that a solute is dissolved in a sea of solvent molecules.

## 3 Conclusion

We determined deuteron quadrupole coupling constants *χ*_D_ and reorientation correlation times *τ*_c_ of imidazolium cations in AAILs, which provide information about the interaction, structure, and dynamics at the molecular level of these biodegradable, nontoxic, and inexpensive compounds. The reorientation correlation times *τ*_c_ of the acidic C(2)─H groups on the imidazolium rings could be obtained from NMR deuteron relaxation times by using an established method for accurately describing *χ*_D_. The obtained *χ*_D_ values for the same C—D bonds in the cation range between 180 and 210 kHz, depending on the differently strong cation–anion interaction further enhanced by hydrogen bonding. The order of the *χ*_D_ values reflect earlier measured vibrational bands in the far infrared region, which were found to shift to higher wavenumbers with enhanced cation–anion interaction. The resulting reorientation correlation times *τ*_c_ indicate that the extreme narrowing condition is fulfilled for this type of ILs. Using the SED relation, the correlation times *τ*_c_ and the additionally measured viscosities *η* provide an estimate for the volume/size of the clusters present in solution. The SED relation seems to be valid only for the IL [EMIm][QAc] wherein we find “quasi” ion pairs due to strong hydrogen bonds between cation and anion and no H─bonding across all ions as for the AAILs.

## Conflict of Interest

The authors declare no conflict of interest.

## Supporting information

Supplementary Material

## Data Availability

The data that support the findings of this study are available from the corresponding author upon reasonable request.

## References

[1] K. Fukumoto , M. Yoshizawa , H. Ohno , J. Am. Chem. Soc. 2005, 127, 2398. 15724987 10.1021/ja043451i

[2] H. Ohno , K. Fukumoto , Acc. Chem. Res. 2007, 40, 1122. 17970600 10.1021/ar700053z

[3] H. Weingärtner , Angew. Chem., Int. Ed. 2008, 47, 654. 10.1002/anie.200604951 17994652

[4] K. Fumino , S. Reimann , R. Ludwig , Phys. Chem. Chem. Phys. 2014, 40, 21903. 10.1039/c4cp01476f 24898478

[5] N. V. Plechkova , K. R. Seddon , Chem. Soc. Rev. 2008, 37, 123. 18197338 10.1039/b006677j

[6] P. A. Hunt , C. R. Ashworth , R. P. Matthews , Chem. Soc. Rev. 2015, 44, 1257. 25582457 10.1039/c4cs00278d

[7] R. Hayes , G. Warr , R. Atkin , Chem. Rev. 2015, 115, 6357. 26028184 10.1021/cr500411q

[8] C. R. Ashworth , R. P. Matthews , T. Welton , P. A. Hunt , Phys. Chem. Chem. Phys. 2016, 18, 18145. 27328990 10.1039/c6cp02815b

[9] S. Grimme , W. Hujo , B. Kirchner , Phys. Chem. Chem. Phys. 2012, 14, 4875. 22378355 10.1039/c2cp24096c

[10] E. I. Izgorodina , Z. L. Seeger , D. L. A. Scarborough , S. Y. S. Tan , Chem. Rev. 2017, 117, 6696. 28139908 10.1021/acs.chemrev.6b00528

[11] K. Fumino , V. Fossog , P. Stange , D. Paschek , R. Hempelmann , R. Ludwig , Angew. Chem., Int. Ed. 2015, 54, 2792. 10.1002/anie.201411509 25639210

[12] R. Ludwig , Phys. Chem. Chem. Phys. 2015, 17, 13790. 25858074 10.1039/c5cp00885a

[13] D. H. Zaitsau , V. N. Emel'yanenko , P. Stange , C. Schick , S. P. Verevkin , R. Ludwig , Angew. Chem., Int. Ed. 2016, 55, 11682. 10.1002/anie.201605633 27504994

[14] D. Kotwica , R. Ludwig , ChemPhysChem 2025, 26, 2500297. 10.1002/cphc.202500297 PMC12503908 40588843

[15] R. Ludwig , F. Weinhold , T. C. Farrar , J. Chem. Phys. 1995, 103, 6941.

[16] M. Strauch , A.‐M. Bonsa , B. Golub , V. Overbeck , D. Michalik , D. Paschek , R. Ludwig , Phys. Chem. Chem. Phys. 2016, 18, 17788. 27067640 10.1039/c6cp01462c

[17] D. T. Emonds , A. L. Mackay , J. Magn. Reson. 1975, 20, 515.

[18] H. Bluyssen , J. Verhoeven , A. Dymanus , Phys. Lett. 1967, 25A, 214.

[19] D. Lankhorst , J. Schriever , J. C. Leyte , Ber. Bunsenges. Phys. Chem. 1982, 86, 215.

[20] R. Ludwig , Chem. Phys. 1995, 195, 329.

[21] P. Stange , S. P. Verevkin , R. Ludwig , Acc. Chem. Res. 2023, 56, 3441. 37956209 10.1021/acs.accounts.3c00530

[22] G. R. Desiraju , T. Steiner , The Weak Hydrogen Bond In Structural Chemistry And Biology, Oxford University Press, Oxford 1999.

[23] M. J. Earle , J. M. S. S. Esperanca , M. A. Gilea , J. N. Canongia Lopes , L. P. N. Rebelo , J. W. Magee , K. R. Seddon , J. A. Widegren , Nature 2006, 439, 831. 16482154 10.1038/nature04451

[24] A. Yokozeki , D. J. Kasprzakb , M. B. Shiflett , Phys. Chem. Chem. Phys. 2007, 9, 5018. 17851598 10.1039/b706497g

[25] A. Wulf , K. Fumino , R. Ludwig , J. Phys. Chem. A 2010, 114, 685. 20014811 10.1021/jp9080146

[26] David S. Raiford , Cherie L. Fisk , Edwin D. Becker , Anal. Chem. 1979, 51, 2050.

[27] E. D. Glendening , J. K. Badenhoop , A. E. Reed , J. E. Carpenter , J. A. Bohmann , C. M. Morales , C. R. Landis , F. Weinhold , NBO 6.0., Theoretical Chemistry InstituteUniversity of Wisconsin, Madison 2013.

[28] F. Weinhold , C. R. Landis , Valency And Bonding A Natural Bond Orbital Donor‐Acceptor Perspective, Cambridge, University Press, Cambridge 2005.

[29] R. Ludwig , F. Weinhold , T. C. Farrar , J. Phys. Chem. A 1997, 101, 8861.

[30] R. Ludwig , F. Weinhold , T. C. Farrar , J. Chem. Phys. 1997, 107, 499.

[31] M. J. Frisch , G. W. Trucks , H. B. Schlegel , G. E. Scuseria , M. A. Robb , J. R. Cheeseman , G. Scalmani , V. Barone , G. A. Petersson , H. Nakatsuji , X. Li , M. Caricato , A. Marenich , J. Bloino , B. G. Janesko , R. Gomperts , B. Mennucci , H. P. Hratchian , J. V. Ortiz , A. F. Izmaylov , J. L. Sonnenberg , D. Williams‐Young , F. Ding , F. Lipparini , F. Egidi , J. Goings , B. Peng , A. Petrone , T. Henderson , D. Ranasinghe , et al. Gaussian 09, Revision D.01, Gaussian, Inc., Wallingford CT 2013.

[32] S. Grimme , J. Antony , S. Ehrlich , H. Krieg , J. Chem. Phys. 2010, 132, 154104. 20423165 10.1063/1.3382344

[33] S. Grimme , S. Ehrlich , L. Goerigk , J. Comput. Chem. 2011, 32, 1456. 21370243 10.1002/jcc.21759

[34] S. Grimme , A. Jansen , Chem. Rev. 2016, 116, 5105. 27077966 10.1021/acs.chemrev.5b00533

[35] H. Huber , J. Chem. Phys. 1985, 83, 4591.

[36] R. Ludwig , F. Weinhold , T. C. Farrar , J. Chem. Phys. 1996, 105, 8223.

[37] J. Busch , R. Ludwig , D. Paschek , J. Phys. Chem. B 2025, 129, 2573. 40001352 10.1021/acs.jpcb.4c08526

[38] K. Fumino , A. Wulf , R. Ludwig , Angew. Chem., Int. Ed. 2008, 47, 8731. 10.1002/anie.200803446 18846527

[39] K. Fumino , T. Peppel , M. Geppert‐Rybczyńska , D. H. Zaitsau , J. K. Lehmann , S. P. Verevkin , M. Köckerling , R. Ludwig , Phys. Chem. Chem. Phys. 2011, 13, 14064. 21666914 10.1039/c1cp20732f

[40] A. Abragam , The Principles Of Nuclear Magnetism, Oxford Clarendon Press 1961.

[41] T. C. Farrar , An Introduction To Pulse NMR Spectroscopy, Farragut Press, Chicago 1987.

[42] H. Weingärtner , Curr. Opin. Colloid Interface Sci. 2013, 18, 183.

[43] A. Wulf , R. Ludwig , P. Sasisanker , H. Weingärtner , Chem. Phys. Lett. 2007, 439, 323.

[44] M. A. Wendt , T. C. Farrar , Mol. Phys. 1998, 95, 1077.

[45] M. A. Wendt , M. D. Zeidler , T. C. Farrar , Mol. Phys. 1999, 97, 753.

[46] T. C. Farrar , T. D. Ferris , M. D. Zeidler , Mol. Phys. 2000, 98, 737.

[47] A. S. L. Gouveia , L. C. Tomé , I. M. Marrucho , J. Chem. Eng. Data 2016, 61, 83.

[48] N. Muhammad , Z. B. Man , M. A. Bustam , M. I. A. Mutalib , C. D. Wilfred , S. Rafiq , J. Chem. Eng. Data 2011, 56, 3157. 10.1007/s12010-011-9315-y 21720837

[49] C. Herrera , G. García , M. Atilhan , S. Aparicio , J. Mol. Liq. 2016, 213, 201.

[50] A. Einstein , Investigations On The Theory Of Brownian Motion, Dover, New York 1956.

[51] P. Debye , Polar Molecules, Dover, New York 1929.

[52] A. Gierer , K. Wirtz , Z. Naturforsch., A: Phys. Sci. 1953, 8, 532.

[53] T. Köddermann , D. Paschek , R. Ludwig , ChemPhysChem 2007, 8, 2464. 17943710 10.1002/cphc.200700552

[54] T. Köddermann , R. Ludwig , D. Paschek , ChemPhysChem 2008, 9, 1851. 18752221 10.1002/cphc.200800102

[55] A. Wulf , K. Fumino , D. Michalik , R. Ludwig , ChemPhysChem 2007, 8, 2265. 17910024 10.1002/cphc.200700508

[56] K. Fumino , A. Wulf , S. P. Verevkin , A. Heintz , R. Ludwig , ChemPhysChem 2010, 11, 1623. 20391460 10.1002/cphc.201000140

