# Supplementary material for: Deuteron Quadrupole Coupling Constants and Reorientation Correlation Times of Cations in Amino Acid Ionic Liquids
Source: Chemphyschem. 2025 Oct 26;26(24):e202500490. doi: 10.1002/cphc.202500490 (PMC12710139; doi:10.1002/cphc.202500490)
Supplement: Supplementary file 1 — Supplementary Material [file CPHC-26-e202500490-s001.pdf]

Deuteron quadrupole coupling constants and reorientation correlation times of cations in amino acid ionic liquids

David Kotwica[a], Dirk Michalik[a,b], Ralf Ludwig[a,b,c]*

a Universität Rostock, Institut für Chemie, Abteilung für Physikalische Chemie, Albert-Einstein-Str. 27, 18059 Rostock, Germany; Tel: 49 381 498 6517; E-mail: ralf.ludwig@uni-rostock.de

b Leibniz-Institut für Katalyse an der Universität Rostock e.V. Albert-Einstein-Str. 29a, 18059 Rostock (Germany)

b Department LL&M, University of Rostock, Albert-Einstein-Str. 25, 18059 Rostock, Germany

Supporting Information (ESI)

Table of contents:

- Sample preparation
- NMR chemical shifts and T_1-relaxation times
- Densities and viscosities
- Computational details
- Chemical shifts of neat IL's and in solution for ^{13}C

Sample preparation and deuteration of Ionic Liquids:

The Ionic liquids have been purchased from IOLITEC and have been characterized by standard NMR spectroscopy and IR spectroscopy. Furthermore 1-Ethyl-3-methylimidazolium glycinate, 1-Ethyl-3-methylimidazolium alaninate and 1-Ethyl-3-methylimidazolium prolinate have been characterized by elemental analysis.

Each sample has been dried thoroughly by a high vacuum membrane pump at a pressure of $2*10^{-5}$ mbar at a temperature of 60 °C for at least one day. Upon drying the samples were stored under nitrogen atmosphere. Prior to each measurement and following preparation steps, the samples were dried again for at least one hour.

Deuteration was ensured by repeatedly purging the sample with sufficient amount of D_2O and drying under inert conditions. The purging was conducted five times to ensure suitable deuteration. At the end of the recycle process the samples were dried like stated above.

NMR chemical shifts and T_1-relaxation times:

The ^{1}H NMR spectra of the Ionic Liquids were recorded on a BRUKER 250 MHz spectrometer using a 5 mm probe. The relaxation times for C(2)-D were measured on the same instrument at 38.37 MHz by using the inversion recovery pulse sequence. Temperature calibrations were carried out using an ethylene glycol NMR thermometer.

Densities and viscosities:

Measurements were conducted with a Anton Paar DMSA 5000M und LOVIS 2000 ME from ANTON PAAR. After initial Drying, the samples were degassed in an ultra-sonic bath.

Computational details:

All density functional theory (DFT) calculations of the IL oligomers have been conducted with the B3LYP method and the 6-31+G* basis set. All optimization steps have been carried out prior the final optimization and frequency calculations. For the utilized structures all convergence criteria have been met (Maximum force, RMS force, Maximum Displacement, RMS displacement). In each calculation empirical dispersion interaction (GD3BJ from the Grimme group) have been included.

For the determination of the gas phase chemical shifts and the electric field gradient of each oligomer the optimized structures were taken and calculated. Obtained calculated values were assigned to each position of interest and matched to the corresponding spectroscopic quantity.

Effective volumes were obtained from the already optimized Ion pair monomers and the single cations and anions.

Chemical shifts of neat IL's and in solution for ^{13}C:

EMIm OAc (**neat**):

(**^{1}H, 298 K, 250 MHz**):

10,24 (s, 1H, C2); **8,12** (s/d, 1H, C5); **7,95** (s/d, 1H, C4); **3,9** (q, 7,3 Hz, 2H); **3,62** (s, 3H); **1,12** (s, 3H); **0,89** (t, 7,3 Hz, 3H)

(**^{13}C, 298 K, DMSO-d6, 62,5 MHz**):

172,92 (**C**OO-); **137,49** (**C**2); **123,42** (**C**4); **121,87** (**C**5); **43,85** (**C**H_2-(CH_3)); **35,40** (-**C**H_3); **26,00** (-**C**H_3, Anion); **15,14** (**C**H_3-(CH_2))

EMIm Gly (**neat**):

(**^{1}H, 298 K, 250 MHz**): **9,80** (s, 1H, C2); **7,77** (s/d, 1,64 Hz, 1H, C5); **7,62** (s/d, 1,64 Hz, 1H, C4); **3,7** (q, 7,3 Hz, 2H); **3,41** (s, 3H); **2,25** (s, 2H) **1,96** (s, 2H, NH_2); **0,67** (t, 7,3 Hz, 3H)

(**^{13}C, 298 K, DMSO-d6, 62,5 MHz**):

172,92 (**C**OO-); **137,18** (**C**2); **123,46** (**C**4); **121,89** (**C**5); **46,32** (**C**H_2, Anion); **43,92** (**C**H_2-(CH_3)); **35,48** (-**C**H_3); **15,13** (**C**H_3-(CH_2))

EMIm Ala (**neat**):

(**^{1}H, 298 K, 250 MHz**): **9,95** (s, 1H, C2); **7,89** (s/d, 1,64 Hz, 1H, C5); **7,72** (s/d, 1,64 Hz, 1H, C4); **3,74** (q, 7,3 Hz, 2H); **3,45** (s, 3H); **2,36** (q, 6,7Hz, 1H); **1,88** (s, 2H, NH_2); **0,70** (t, 7,3 Hz, 3H); **0,33** (d, 6,7 Hz, 3H)

(**^{13}C, 298 K, DMSO-d6, 62,5 MHz**):

177,99 (**C**OO-); **137,35** (**C**2); **123,44** (**C**4); **121,86** (**C**5); **51,89** (**C**H_3, Anion); **43,92** (**C**H_2-(CH_3)); **35,48** (-**C**H_3); **22,92** (**C**H, Anion); **15,16** (**C**H_3-(CH_2))

EMIm Pro (**neat**):

(**^{1}H, 298 K, 250 MHz**): **9,94** (s, 1H, C2); **7,86** (s/d, 1,64 Hz, 1H, C5); **7,68** (s/d, 1,64 Hz, 1H, C4); **3,72** (q, 7,3 Hz, 2H); **3,42** (s, 3H); **2,21** (m, 1H); **1,84** (m, 2H); **1,05** (m, 2H); **0,92** (m ,2H); **0,71** (t, 7,3 Hz, 3H)

(**^{13}C, 298 K, DMSO-d6, 62,5 MHz**):

176,72 (**C**OO-); **137,14** (**C**2); **123,45** (**C**4); **121,88** (**C**5); **62,86** (**C**α, Anion); **47,16** (**C**δ, Anion); **43,92** (**C**H_2-(CH_3)); **35,47** (-**C**H_3); **31,27** (**C**β, Anion); **26,18** (**C**γ, Anion); **15,13** (**C**H_3-(CH_2))
